# Effects of Anonymity versus Examinee Name on a Measure of Depressive Symptoms in Adolescents

**DOI:** 10.3390/children9070972

**Published:** 2022-06-29

**Authors:** César Merino-Soto, Anthony Copez-Lonzoy, Filiberto Toledano-Toledano, Laura A. Nabors, Jorge Homero Rodrígez-Castro, Gregorio Hernández-Salinas, Miguel Ángel Núñez-Benítez

**Affiliations:** 1 Instituto de Investigación en Psicología, Universidad de San Martin de Porres, Av. Tomas Marsano 342, Lima 34, Peru; sikayax@yahoo.com.ar; 2 Unidad de Investigación en Bibliometría, Universidad San Ignacio de Loyola, Av. la Fontana 750, Lima 12, Peru; anthonycopez22@gmail.com; 3 Unidad de Investigación en Medicina Basada en Evidencias, Hospital Infantil de México Federico Gómez National Institute of Health, Dr. Márquez 162, Doctores, Cuauhtémoc, Mexico City 06720, Mexico; 4 Unidad de Investigación Sociomédica, Instituto Nacional de Rehabilitación Luis Guillermo Ibarra Ibarra, Calzada México-Xochimilco 289, Arenal de Guadalupe, Tlalpan, Mexico City 14389, Mexico; 5 School of Human Services, College of Education, Criminal Justice and Human Services, University of Cincinnati, Cincinnati, OH 45221-0068, USA; naborsla@ucmail.uc.edu; 6 Tecnológico Nacional de Mexico, Instituto Tecnológico de Ciudad Victoria, División de Estudios de Posgrado e Investigación, Boulevard Emilio Portes Gil #1301 Pte. A.P. 175 C.P., Ciudad Victoria 87010, Mexico; jorge.rc@cdvictoria.tecnm.mx; 7 Tecnológico Nacional de México/Instituto Tecnológico Superior de Zongolica-Extensión Tezonapa, Km. 4 Carr. a La Compañia S/N, Tepetitlanapa, Zongolica 95005, Mexico; gregorio_18_18@live.com.mx; 8 Unidad de Medicina Familiar 31, Ermita Iztapalapa 1771, 8va Amp San Miguel, Iztapalapa, Mexico City 09837, Mexico; aquetzalli03@yahoo.com.mx

**Keywords:** adolescents, assessment, anonymous response, validity, depression measure

## Abstract

There is evidence in the literature that anonymity when investigating individual variables could increase the objectivity of the measurement of some psychosocial constructs. However, there is a significant gap in the literature on the theoretical and methodological usefulness of simultaneously assessing the same measurement instrument across two groups, with one group remaining anonymous and a second group revealing identities using names. Therefore, the aim of this study was to compare the psychometric characteristics of a measure of depressive symptoms in two groups of adolescents as a consequence of identification or anonymity at the time of answering the measuring instrument. The participants were 189 adolescents from Metropolitan Lima; classrooms were randomly assigned to the identified group (n = 89; application requesting to write one’s own name) or to the anonymous group (n = 100; application under usual conditions), who responded to the Childhood Depression Inventory, short version (CDI-S). Univariate characteristics (mean, dispersion, distribution), dimensionality, reliability, and measurement invariance were analyzed. Specific results in each of the statistical and psychometric aspects evaluated indicated strong psychometric similarity. The practical and ethical implications of the present results for professional and research activity are discussed.

## 1. Introduction

According to systematic reviews on the topic of social desirability in the clinical context, a situation that represents a pervasive risk in applied research based on self-report, application for clinical and forensic practice seems to be attributed to the identification of those evaluated [1,2]. To mitigate response biases associated with self-report and to conform to ethical standards, participants are generally asked to respond anonymously. The requirement of response anonymity has a long history in the application of surveys of all types, as well as its implications for its adequacy in the ethical standards of scientific research. However, this strategy is also exposed to particular effects due to its subjective value on the privacy of responses [3], and anonymity has influenced the quality of responses even in ethnocultural research contexts and clinical applications, overestimating scores [4,5,6]. On the other hand, in nonclinical samples, anonymity may reduce the sense of responsibility in the response process [7], even more so in the face of stigmatizing behaviors such as stealing, cheating, psychoactive substance use, and erotophilic behaviors [8,9,10,11,12]. Even in circumstances of respondent quasi-identity, perceived identity protection influences the possible contamination of scores related to gender, age, and place of origin [9]. Finally, response anonymity does not guarantee the absence of careless responses or insufficient effort (C/IE; [13]), given that this type of response is almost always present in anonymous surveys presented in pencil–paper format or on a web platform [13,14,15].

At this point, we arrive at the following question: Would there be an impact on mental health assessments of adolescents if their responses are anonymous or identified? Apparently, this question has not been asked before in the context of screening the adolescent community, and it seems possible that it has not been asked in published research. The anonymous responses of adolescents in assessments for research purposes does not appear to be problematic because no actual or masked identification of the evaluee is usually required; however, in screening assessments for emotional problems within an institution, accurate referral requires identifying the adolescent being assessed to refer him or her to appropriate clinical intervention services [16,17]. The identification of the symptomatology associated with childhood depression is essential to reduce its effects on the mental health of children and adolescents. The USA’s National Institute of Mental Health [18] indicated that in the preadult stage (among children and adolescents), approximately three million individuals suffer from mental disorders [18] and require proper identification to provide them with timely clinical services. This is more sensitive because the adolescent stage is vulnerable to mood alterations, social and school behavioral changes, and transition to new family roles [19,20]. Therefore, in the context of the assessment of children and adolescents, it is necessary to use screening instruments that are widely applied [17] and to use especially short scales, because they reduce irrelevant variance, potentially producing acceptable levels of specificity and sensitivity and thus improving control of Type I and Type II errors in identification and referral to clinical services [21,22,23,24,25]. Like longer measures with more items, short scales have advantages and limitations that the user must weigh out in deciding on their use and the interpretation of their scores. However, in the context of mass use and given the purpose of screening, short scales with good evidence of validity may be the best option.

One of the instruments for the detection of childhood depression is the Child Depression Inventory (CDI; [26]). This measure has a shorter version (Child Depression Inventory-Short, CDI-S) that is used as a screening and treatment follow-up instrument. It takes between 5 and 10 min to administer and even less time to score. Overall, the CDI-S is a cost- and speed-efficient tool for assessing behaviors of low population prevalence [27] and for the assessment of adults with intellectual disabilities [28] and populations with physical disabilities [29], including in the school context [30]. The CDI-S can have better evaluative efficiency than the full version because of its intrinsic and psychometric characteristics, and one of them is its dimensionality. That is, while studies using the full version yield different factorial solutions (between three and eight factors, possibly associated with the analysis strategies applied and the criteria that the different authors applied; [27]), the dimensionality of the brief version seems less problematic due to the reduced number of items. If the internal structure of the CDI is modified in subsequent studies, the problem lies in the instability (a) of the construct to be generalizable across contexts and (b) of the content sampling of the construct as originally planned. Additionally, whenever this structure of the instrument changes, the interpretation is not always statistically or conceptually comparable across groups or studies.

The CDI-S uses the self-report method, and this type of procedure generally presents several challenges that the researcher or clinician must recognize and address. For example, one such limit is social desirability [3,8,12], which tends to interact with the examinee’s perception of threat toward the evaluative situation [31,32]. Therefore, the aim of this research was to analyze the effect of anonymity and subject identification on the psychometric properties (internal structure, reliability, measurement invariance) of the CDI-S scores in the adolescent population. This was conducted in the context of the natural application of screening instruments to detect early symptoms of depression.

## 2. Materials and Methods

### 2.1. Participants

In total, 189 Peruvian adolescent students living in Metropolitan Lima were enrolled. All were enrolled in a public, tuition-free educational institution at the secondary elemental level. The majority (69.1%) lived in the same district in which the institution was located. The mean age was 13.23 and ranged from 11 to 17 years (SD = 1.14). The distribution of students in their grades of study was as follows: first (51, 26.0%), second (64, 32.7%), third (69, 35.2%), and fourth (12, 6.1%). The demographic characteristics of the adolescents are shown in Table 1. For the purposes of the study, the participants were divided into two groups, using the classroom as the unit to identify them and randomly assign the modification of the CDI-S filling instructions (see the Procedure section); the groups were identified as group A (those who received the unmodified instructions, n = 100) and group B (modified group, or those who received the modified instructions, n = 89).

### 2.2. Instruments

Children’s Depression Inventory-Short (CDI-S; [33]). The Spanish version of the CDI-S [34] was used. This self-report is used to screen for depressive symptoms, derived from the 27 item long version. The CDI-S can be applied to children and adolescents between the ages of 7 and 17 years individually or in groups. The CDI-S consists of 10 items selected by the author as the most representative of the construct, and its format is identical to that of the longer version. Each item has three phrases describing symptoms ranging from less (absence of the symptom) to more intense (severe presence of the symptom). The instructions ask the participants to choose the sentence that best fits how he or she has felt in the last 15 days. Items 2, 4, 5, 6, and 10 are reverse scored. The internal consistency coefficient found in the adaptation of the Hispanic version was 71 [34].

### 2.3. Ethical Considerations

This study is a part of the research project (HIM/2015/017/SSA.1207; “Effects of mindfulness training on psychological distress and quality of life of the family caregiver”) that was approved on 16 December 2014 by the Research, Ethics, and Biosafety Commissions of the Hospital Infantil de México Federico Gómez National Institute of Health in Mexico City. While conducting this study, the ethical rules and considerations for research with humans currently enforced in Mexico [35] and those outlined by the American Psychological Association [36] were followed. All family caregivers were informed of the objectives and scope of the research and their rights in accordance with the Declaration of Helsinki [37]. The caregivers who agreed to participate in the study signed an informed consent letter. Participation in this study was voluntary and did not involve payment. The caregivers who provided consent for their child to participate completed an informed consent letter. Youth participants provided assent and returned a survey if they wished to participate.

### 2.4. Procedure

The authorization of the directors of the educational institution was obtained, and the corresponding permissions were requested from the parents, who were informed of the research proposal and the data collection procedures. Once the directors and parents agreed to participate, the instrument was administered during class time. The students who provided assent completed the CDI-S. Classrooms were randomly assigned to groups A and B; these groups had different instructions for filling out the CDI-S: group “A” received instructions to fill out the CDI-S anonymously, while group “B” was asked to give their name in order to have a better identification at the time of collecting the completed questionnaires. The general instruction given to the adolescents emphasized that they could stop responding at any time, without consequence. All information on examinees in both groups was transferred to a database, but the names of the examinees in group B were not entered into this database. When the database was completed, the written names were removed from the paper questionnaires.

### 2.5. Data Analysis

The analysis consisted of univariate and multivariate analysis phases. First, several statistical aspects of the items, such as distribution, location (mean), dispersion (standard deviation), and floor and ceiling (minimum and maximum frequency of response), were analyzed. The statistical comparison between the distributions of each item was made using the KS-D test [38,39] for two independent samples, and the overlap coefficient (OVL; [40]) was used as a measure of the practical significance of the comparison of two distribution functions that are not necessarily normally distributed [41]; the model was used for different variances to ensure better precision.

The internal structure of the CDI-S was examined by a confirmatory factor analysis, with the maximum likelihood method adjusted for item nonnormality (SB-χ^2^; [42]), on the matrix of interitem polychoric correlations; given the limited number of response categories, this approach can be a satisfactory estimation method [43,44,45]. The measurement invariance of the items was examined by means of two procedures: the first was the metric congruence of the items [46], in which the factor loadings of the items of each group were compared by means of the congruence coefficient (φ; [46]). The second procedure used differential item functioning analysis (DIF; [47]), with the following specifications: (a) the matching variable was the observed score, θ, and (b) the grouping variable (G) was the status of the group examined, where the reference group was “A” (anonymous group) and the focus group was “B” (provided names). The DIF analysis was implemented with ordinal logistic regression (OLR; [48]), in which each item was assumed to be a dependent and continuous latent variable (Z, standardized in logits). The independent variables were the measured attribute (or observed score, θ), subject grouping (G; group A vs. group B), and attribute–group interaction (θ*G). Each represents a different type of DIF [49,50]. The OLR methodology consists of modeling three equations: one representing the nonuniform DIF (OLR_1_, Z = *β*_0_ + *β*_1_θ + *β*_2_G + *β*_3_θ*G), one for uniform DIF (OLR_2_, Z = *β*_0_ + *β*_1_θ + *β*_2_G), and another model for representing responses without DIF (OLR_3_, Z = *β*_0_ + *β*_1_θ). The stepwise screening strategy [49,50] focused on the evaluation of practical and statistical significance, according to which for each item we first evaluated the difference between the −2 log likelihood (Δ*χ*^2^, gl = 1, *α* = 0.05) between OLR_1_ and OLR_2_ models for detection of no uniform DIF (null hypothesis: OLR_1_ = OLR_2_) and then between OLR_3_ and OLR_2_ for detection of uniform DIF. The Bonferroni correction [50,51] was applied to adjust nominal *α* according to the number of items (0.05/10 = 0.005). Results below this level (*α_Bonferroni_ =* 0.005) identified the impact of the interaction term (θ*G) and therefore the presence of nonuniform DIF. If the previous null hypothesis (nonuniform DIF) is not rejected, the second step tested the uniform DIF by the difference (Δ) of the beta coefficients of the models OLR_3_ (*β*_θ_) and OLR_2_ (*β*_G_). A result ≥10% indicated statistical significance at the nominal level *α* = 0.20 [50].

Finally, reliability was estimated by the *α* coefficient [52] and *ω* [53]; although *ω* tends to be more appropriate [54], the *α* coefficient was also reported because it is a measure of score reliability that (a) is still popular in behavioral science research, (b) serves for direct comparison with the Spanish validation study, and (c) allows for comparison with the ω coefficient to assess the impact of possible noncompliance with the basic assumption for using α [54].

## 3. Results

### 3.1. Equivalence between Groups

The equivalence of characteristics in both groups was analyzed in an equivalence testing framework [55]. To maximize the sensitivity of the test for equivalence of means, the minimum standardized difference was set at *d* = 0.10 [56]. This showed that the average ages of the two groups were equivalent, *t* = 0.90 (gl = 194, *p* = 0.40). Similarly, statistical and practical equivalence between the two groups was found in the distribution of the following variables: school levels, Mantel–Haenszel χ^2^ (gl: 1) = 3.20, p = 0.07, γ = 0.178; sex, χ^2^ (gl: 1)= 5.86, p = 0.48, V_Cramer_ = 0.05; family configuration, χ^2^ (gl: 2) = 5.86, p = 0.05, V_Cramer_ = 0.17; place of birth, χ^2^ (gl: 1) = 1.61, p = 0.20, V_Cramer_ = 0.09; mother’s level of education, Mantel–Haenszel χ^2^ (gl: 1) = 1.80, p = 0.17, V_Cramer_ = 0.16; and father’s level of education, Mantel–Haenszel χ^2^ (gl: 1) = 0.4, p = 0.83, V_Cramer_ = 0.13. Considering these results, the sociodemographic equivalence of both groups can be accepted.

### 3.2. Univariate Analysis

#### 3.2.1. Items Level

**Figure 1:**
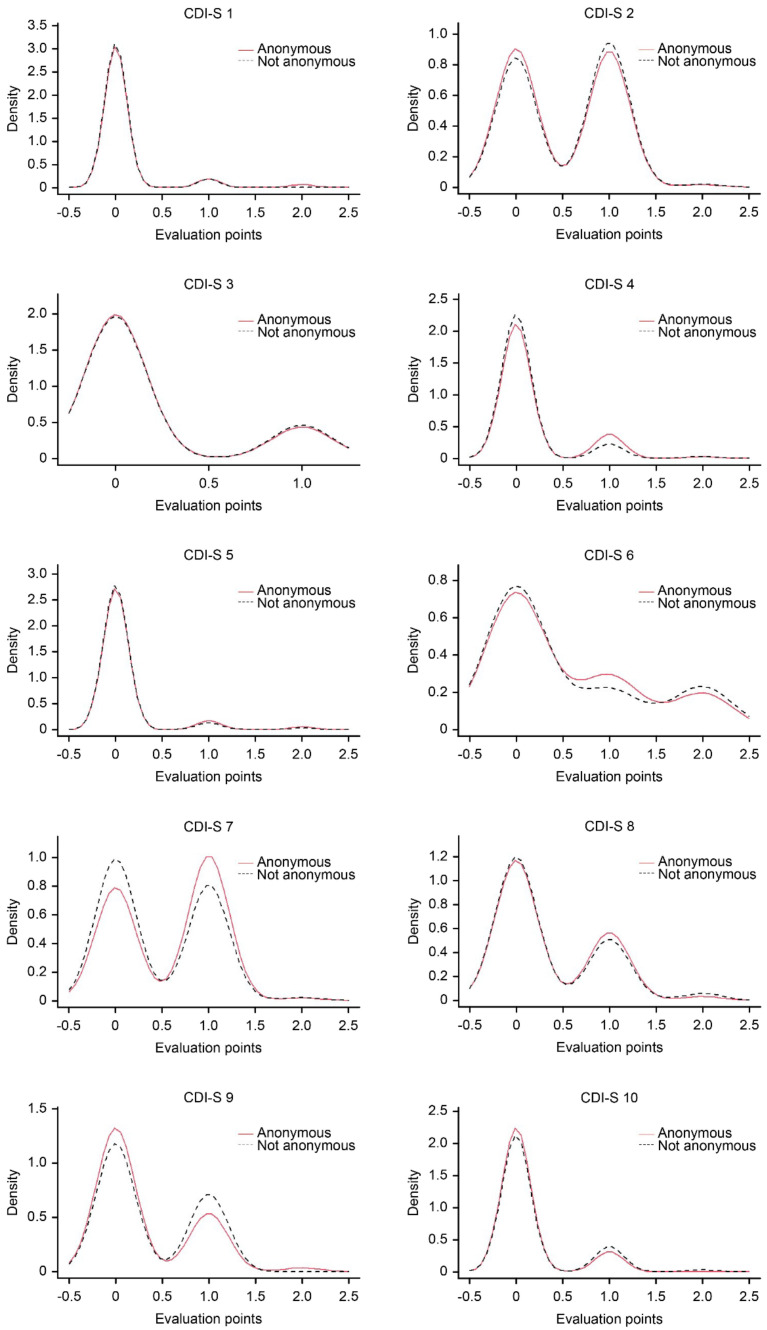
Comparative density distribution of responses to items.

The univariate statistics for the items (Table 2) in both groups were similar, and the discrepancies can be established as small. The Pearson correlations of these descriptive statistics (M, SD, *g*_1_, *g*_2_, floor and ceiling effect) between groups A and B were 0.96, 0.94, 0.95, 0.92, 0.95, and 0.97, respectively. These high magnitudes confirm that the pattern of descriptive statistics at the item level was similar between the compared groups. To verify this more rigorously, the statistics for each item were analyzed individually. In Table 3, the distributional differences in the response range of each item were not statistically significant (KS-D between 0.009 and 0.11), and the degree of overlap (coefficient OVL) between the distributions was greater than 79.2% but approximately 95%, suggesting that the items showed practically overlapping distributions between groups A and B (see Figure 1). Differences in the location or media (*d* between |0.000| and |0.210|; *t*-test < 1.50) and variances (*F_L_*; Levene [57]; α nominal with Bonferroni correction: 0.05/10 = 0.005) were essentially trivial and not statistically significant. These results, taken together, point to univariate similarity at the item level between the two groups.

#### 3.2.2. Score Level

Descriptive results in groups A (*M* = 2.981, *SD* = 2.629) and B (*M* = 2.900, *SD* = 2.312) suggested essential similarity (*t* = 0.23, gl = 194, *p* = 0.185), which was verified with a mean equivalence analysis [55] and contrasted against a standardized difference *d* = 0.10. The difference between variances (robust test, *F_L_*; [57]) also pointed to insubstantial differences (*F_L_* = 1.170, *p* = 0.281).

### 3.3. Internal Structure

#### 3.3.1. Dimensionality

The fit of the items to a unidimensional model was good (*p* > 0.05, df = 35) for both groups: group A, SB-*χ*^2^ = 34.63, CFI = 1.00, RMSEA = 0.00 (90% CI = 0.00, 0.07); group B, SB-*χ*^2^ = 36.11, CFI = 0.99, RMSEA = 0.01 (90% CI = 0.00, 0.07). Within each dimension, factor loadings were high but heterogeneous (see Table 2, CFA heading).

#### 3.3.2. Differential Item Functioning: Anonymity vs. Examinee Name

The metric congruence (equality of factor loadings between groups A and B) was φ = 0.989, which presents substantial equality between them [46]. The analysis of the nonuniform and uniform DIF (Table 4) in each item showed the absence of any type of DIF.

#### 3.3.3. Reliability

The *α* coefficients for groups A and B were 0.741 and 0.633, respectively; the difference between them [58] was not statistically significant, *W* = 1.417, *F*(93, 81) = 1.43: ω coefficients were 0.898 and 0.835 for groups A and B, respectively, and can also be considered to be similar.

## 4. Discussion

The aim of this research was to analyze the effect of anonymity and identification by name of adolescents in a research context. This effect was examined on the statistical and psychometric properties (internal structure, reliability, measurement invariance) of the scores of an abbreviated measure of depressive symptoms, the CDI-S. The findings of the present study are interesting and indicate that there are no effects on the psychometric properties and, consequently, on the interpretation of the CDI-S score. It can be stated that the Type I or II error that could be present in the identification and referral decisions with the CDI-S would probably be less associated with the identification of the assessed person so that the scores obtained are valid. Although a reduction in internal consistency was observed, this was statistically trivial and possibly without relevant effects on the standard error of measurement. This trivial effect was observed mainly for the coefficient *ω,* while the *α* coefficient showed a comparatively smaller reduction. It is possible that this difference interacted with one of the assumptions of coefficient *α*, which is tau equivalence and correlated errors [52], but correlated errors were not detected in the modeling of the dimensionality of the CDI-S in either group. Even with this reduction in internal consistency as measured by the α coefficient, the lack of statistical significance suggests that it may be considered sampling error. A complementary finding is that, in contrast to the Hispanic study by del Barrio et al. [34], here, a single latent dimension was endorsed to the CDI-S and had higher reliability; differences in reliability estimates obtained from two coefficients were also detected (*α* and *ω*), which usually represent noncompliance with the tau-equivalence model in the items [54].

The practical implications of the present results point to several potential consequences. First, the clinician using mass screening strategies now has evidence that respondent identification has a trivial effect on score variability. Second, and as a consequence of the above, the clinician can be confident that CDI-S results are possibly less influenced by subject anonymity or identification. Finally, another no less important implication is of an ethical nature; in this context, the clinician must pay attention to the safety of the tests applied and to the identification of respondents. Within an effective strategy to prevent unauthorized dissemination of the test applied and its results, identification of the examinee creates a more challenging situation than anonymous application.

The results should be interpreted in consideration of the specific limitations of the study. First, the sample size in each group limits its statistical power in each of the statistics applied, but even more so in the representativeness of the population to which it can be generalized. This limitation requires a replication study as a necessary condition to verify this effect of anonymity/identification in survey for schooled adolescents, an issue that apparently has not been addressed in previous studies [9]. On the other hand, a balance for this limitation on sample size is that the robust method used here [42] has proven to be effective in estimating the parameters of interest (the factor loadings) and their statistical significance in challenging situations such as small sample size and distributional skewness of the items [43,44,45]. Therefore, the problem of the accuracy of the estimates may have been partially solved. Second, the sample size also prevented further partitioning along of the main study variable (group A and group B), as it meant further reducing the samples compared; for example, the difference between males and females was not examined in interaction with the effect of anonymity, and the extent to which they affect response variability is not known.

With respect to sample size, previous studies have suggested that by applying multiple criteria (absolute number of cases according to expert opinion, the ratio number of cases—number of parameters or number of observed variables, and statistical power), the range of minimum sample sizes varies from 16 to 2760 cases [59]. Other more sophisticated methods also produce divergence (e.g., on the basis of statistical power; [60,61]). Methodological research has shown that aspects such as the size of factor loadings, communality, and the number of dimensions [62,63] are stable criteria. On the basis of opportunity and contextual constraints in the present study and the minimum sample size for estimating the parameters of interest (e.g., factor loadings and communalities; [62,63]), our sample size may be sufficient (approximately 200).

## 5. Conclusions

The present study shows support for a unidimensional internal structure of the CDI-S in Peruvian adolescents. When specific conditions were imposed on the selection groups (anonymity/identification of the participants), no significant differences were found at the level of internal structure, and therefore both models were acceptable. The CDI-S can be considered a unidimensional measure for use in the general adolescent population (as is the case in our study) since experiencing some condition of dysphoria and/or negative self-esteem does not seem to be differentiable if there is no additive exposure to some clinical condition (e.g., institutionalization, chronic noncommunicable diseases, terminal illnesses); similar findings have been obtained in the literature with other depression assessment instruments (e.g., PHQ-9). Measurement invariance was corroborated, which would imply that the possible impact of anonymity would be closely related to socially inappropriate behaviors. The reliability of the CDI-S scores for both groups was not compromised. This would imply that the measurement bias would not be directly related to an identification condition but rather to other factors already identified in the literature. Due to sample size limitations in the groups of interest, further research is required on other conditions of intergroup variability, such as some sociodemographic variables and mental and physical health conditions versus each condition of anonymity vs. participant name.

## Figures and Tables

**Table 1:** Description of participants (n = 196).

	N	%
Sex		
Male	99	50.5
Female	97	49.5
Place of birth		
Lima	165	84.2
Other	31	15.8
Family structure		
I live with both parents	122	62.2
I live with one of my parents	60	30.6
I live with other people	14	7.1
Mother’s (father’s) education		
Less than high school	30 (25)	15.3 (12.8)
Completed high school	91 (81)	46.4 (41.3)
Technical education (1 to 2 years)	20 (13)	10.2 (10.2)
Technical (3 years)	10 (45)	5.1 (6.6)
University	41 (12)	20.9 (23.0)
No information	4.0 (2.0)	2.0 (6.1)

**Table 2:** Univariate descriptive and CFA for the items CDI-S.

Item	Descriptive Statistics								CFA	
	M	95% CI	SD	*g*_1_	*g*_2_	*r_itc_*	Floor	Ceiling	λ	*h* ^2^
Group A (anonymous)										
Item 1	0.094	[0.01; 0.18]	0.353	4.032	16.751	0.43	92.5	1.90	0.740	0.548
Item 2	0.509	[0.38; 0.64]	0.521	0.168	−1.51	0.396	50	0.90	0.604	0.365
Item 3	0.179	[0.08; 0.28]	0.385	1.697	0.895	0.455	82.1	0.00	0.722	0.521
Item 4	0.17	[0.07; 0.27]	0.402	2.224	4.229	0.496	84	0.90	0.792	0.628
Item 5	0.094	[0.01: 0.18]	0.353	4.032	16.751	0.489	92.5	1.90	0.824	0.679
Item 6	0.557	[0.37: 0.74]	0.757	0.946	−0.603	0.313	60.4	16.0	0.377	0.142
Item 7	0.575	[0.45; 0.70]	0.515	−0.097	−1.505	0.235	43.4	0.90	0.448	0.201
Item 8	0.358	[0.23; 0.49]	0.52	1.009	−0.128	0.614	66	1.90	0.794	0.631
Item 9	0.321	[0.19; 0.45]	0.508	1.217	0.407	0.414	69.8	1.90	0.647	0.419
Item 10	0.123	[0.04; 0.20]	0.33	2.334	3.513	0.431	87.7	0.00	0.810	0.656
Group B (name of examinee)										
Item 1	0.056	[−0.01; 0.12]	0.23	3.947	13.884	0.212	94.4	0.00	0.617	0.380
Item 2	0.544	[0.40; 0.69]	0.523	0.06	−1.462	0.475	46.7	1.10	0.601	0.362
Item 3	0.189	[0.08; 0.30]	0.394	1.617	0.627	0.247	81.1	0.00	0.575	0.330
Item 4	0.111	[0.02; 0.21]	0.35	3.289	11.138	0.453	90	1.10	0.757	0.574
Item 5	0.067	[−0.01; 0.15]	0.292	4.814	24.931	0.443	94.4	1.10	0.876	0.768
Item 6	0.556	[0.34; 0.77]	0.795	0.981	−0.692	0.086	63.3	18.9	0.156	0.024
Item 7	0.467	[0.33; 0.61]	0.524	0.376	−1.331	0.316	54.4	1.10	0.521	0.271
Item 8	0.356	[0.21; 0.50]	0.547	1.233	0.577	0.455	67.8	3.30	0.580	0.336
Item 9	0.378	[0.25; 0.51]	0.488	0.513	−1.777	0.296	62.2	0.00	0.323	0.104
Item 10	0.178	[0.07; 0.29]	0.413	2.193	4.15	0.35	83.3	1.10	0.685	0.469

*g*_1_ and *g*_2_: skewness and kurtosis coefficients; *r_itc_*: item–test correlation; CFA: factor loadings (λ) of the confirmatory factor analysis; *h*^2^: squared factor loading.

**Table 3:** Comparative results between groups.

Item	Distribution		Location (Mean)		Dispersion
	KS-D	OVL	*t* (df)	*d* (95% CI)	*F_L_* (1, 194)
Item 1	0.019	0.792	0.71 (182.440)	0.099 (−0.18; 0.38)	3.363
Item 2	0.033	0.973	−0.40 (188.645)	−0.057 (−0.34; 0.22)	0.007
Item 3	0.009	0.986	−0.18 (187.401)	−0.025 (−0.31; 0.26)	0.119
Item 4	0.060	0.913	1.12 (193.870)	0.150 (−0.12; 0.44)	4.385
Item 5	0.019	0.904	0.43 (193.878)	0.061 (−0.22; 0.34)	1.388
Item 6	0.029	0.976	0.009 (185.561)	0.000 (−0.28; 0.28)	0.393
Item 7	0.110	0.916	1.48 (187.786)	0.210 (−0.07; 0.49)	0.272
Item 8	0.017	0.975	0.038 (185.438)	0.000 (−0.28; 0.28)	0.006
Item 9	0.075	0.951	−0.84 (191.038)	−0.120 (−0.40; 0.16)	0.660
Item 10	0.044	0.880	−1.11 (169.440)	−0.160 (−0.44; 0.12)	4.515

KS-D: D Kolmogorov–Smirnov statistic; OVL: overlap coefficient; *t* (df): Student’s *t*-test for comparison of means and degrees of freedom; *d*: standardized difference; *F_L_*: Levene’s F test (gl_1_, gl_2_).

**Table 4:** Results of differential functioning of items (ordinal logistic regression).

Item	Non-Uniform DIF		Uniform DIF	
	P_Dif.(LL)_	DIF	*β*_1_–*β*_2_	DIF
Item 1	0.226	No	−0.0067	No
Item 2	0.224	No	−0.0032	No
Item 3	0.461	No	0.0075	No
Item 4	0.363	No	−0.0044	No
Item 5	0.981	No	−0.0053	No
Item 6	0.918	No	0.0007	No
Item 7	0.278	No	0.0258	No
Item 8	0.778	No	−0.0000	No
Item 9	0.712	No	0.0057	No
Item 10	0.702	No	0.0517	No

P_Dif. (LL)_: *p* value of difference between the −2 log likelihood; DIF: differential functioning of items.

## Data Availability

The raw data supporting the conclusions of this article will be made available by the authors, without undue reservation.

## References

[1] Perinelli E., Gremigni P. (2016). Use of social desirability scales in clinical psychology: A systematic review. J. Clin. Psychol..

[2] van de Mortel T.F. (2008). Faking it: Social desirability response bias in self-report research. Aust. J. Adv. Nurs..

[3] Whelan T.J. Anonymity and confidentiality: Do survey respondents know the difference?. Proceedings of the 30th Annual Meeting of the Society of Southeastern Social Psychologists.

[4] Ash P., Abramson E. (1952). The effect of anonymity on attitude-questionnaire response. J. Abnorm. Soc. Psychol..

[5] Kraus J. (1975). Effect of anonymity on response of adoptive parents to a child-problems questionnaire. Aust. Soc. Work.

[6] Rosen N.A. (1960). Anonymity and Attitude Measurement. Public Opin. Q..

[7] Lelkes Y., Krosnick J.A., Marx D.M., Judd C.M., Park B. (2012). Complete anonymity compromises the accuracy of self-reports. J. Exp. Soc. Psychol..

[8] Beatty J.R., Chase S.K., Ondersma S.J. (2014). A randomized study of the effect of anonymity, quasi-anonymity, and certificates of confidentiality on postpartum women’s disclosure of sensitive information. Drug Alcohol Depend..

[9] Caballero R., Sen S., Nygård J.F. (2017). Anticipating anonymity in screening program databases. Int. J. Med. Inform..

[10] Durant L.E., Carey M.P., Schroder K.E. (2002). Effects of anonymity, gender, and erotophilia on the quality of data obtained from self-reports of socially sensitive behaviors. J. Behav. Med..

[11] O’Malley P.M., Johnston L.D., Bachman J.G., Schulenberg J. (2000). A Comparison of confidential versus anonymous survey procedures: Effects on reporting of drug use and related attitudes and beliefs in a national study of students. J. Drug Issues.

[12] Ong A.D., Weiss D.J. (2000). The impact of anonymity on responses to sensitive questions. J. Appl. Soc. Psychol..

[13] Meade A.W., Craig S.B. (2012). Identifying careless responses in survey data. Psychol. Methods.

[14] Schneider S., May M., Stone A.A. (2018). Careless responding in internet-based quality of life assessments. Qual. Life Res..

[15] Ward M.K., Meade A.W. (2018). Applying social psychology to prevent careless responding during online surveys. Appl. Psychol..

[16] Martínez I.M.M. (2001). Efectos del anonimato en la comunicación de grupos que utilizan tecnologías asistidas por ordenador. Un estudio cuantitativo y cualitativo. An. Psicol. Ann. Psychol..

[17] Matthey S., Petrovski P. (2002). The children’s depression inventory: Error in cutoff scores for screening purposes. Psychol. Assess..

[18] National Institute of Mental Health (2016). Major Depression among Adolescents.

[19] Bould H., Araya R., Pearson R.M., Stapinski L., Carnegie R., Joinson C. (2014). Association between early temperament and depression at 18 years. Depress. Anxiety.

[20] Garber J., Brunwasser S.M., Zerr A.A., Schwartz K.T., Sova K., Weersing V.R. (2016). Treatment and prevention of depression and anxiety in youth: Test of cross-over effects. Depress. Anxiety.

[21] Emons W.H., Sijtsma K., Meijer R.R. (2007). On the consistency of individual classification using short scales. Psychol. Methods.

[22] Kruyen P.M., Emons W.H.M., Sijtsma K. (2012). Test length and decision quality in personnel selection: When is short too short?. Int. J. Test..

[23] Kruyen P.M., Emons W.H.M., Sijtsma K. (2013). On the shortcomings of shortened tests: A literature review. Int. J. Test..

[24] Kruyen P.M., Emons W.H.M., Sijtsma K. (2014). Assessing individual change using short tests and questionnaires. Appl. Psychol. Meas..

[25] Ziegler M., Kemper C., Kruyen P. (2014). Short scales—five misunderstandings and ways to overcome them. J. Individ. Differ..

[26] Kovacs M. (1985). The children’s depression inventory (CDI). Psychopharmacol. Bull..

[27] Volpe R.J., DuPaul G.J., Andrews J., Janzen H., Saklofske D. (2001). Assessment with brief behavior rating scales. Handbook of Psychoeducational Assessment: Ability, Achievement, and Behavior in Children.

[28] Ailey S.H., Marks B., Heller T. (2003). Evaluation of two self-report depression measures for adults with Down syndrome. NADD Bull..

[29] de la Vega R., Racine M., Sánchez-Rodríguez E., Solé E., Castarlenas E., Jensen M.P., Engel J., Miró J. (2016). Psychometric properties of the short form of the Children’s Depression Inventory (CDI-S) in young people with physical disabilities. J. Psychosom. Res..

[30] Davanzo P., Kerwin L., Nikore V., Esparza C., Forness S., Murrelle L. (2004). Spanish translation and reliability testing of the Child Depression Inventory. Child Psychiatry Hum. Dev..

[31] Holden R.R., Magruder C.D., Stein S.J., Sitarenios G., Sheldon S. (1999). The effects of anonymity on the holden psychological screening inventory. Pers. Individ. Differ..

[32] Sloan D.M., Marx B.P., Epstein E.M., Lexington J.M. (2007). Does altering the writing instructions influence outcome associated with written disclosure?. Behav. Ther..

[33] Sitarenios G., Kovacs M., Maruish M.E. (1999). Use of the children’s depression inventory. The Use of Psychological Testing for Treatment Planning and Outcomes Assessment.

[34] del Barrio V., Roa M.L., Olmedo M., Colodrón F. (2002). Primera adaptación del CDI- S en población española. Acción Psicol..

[35] Sociedad Mexicana de Psicología (2010). Código Ético del Psicólogo [Ethical Code of the Psychologist].

[36] American Psychological Association (2017). Ethical Principles of Psychologists and Code of Conduct. With the 2016 Amendment to Standard 3.04.

[37] World Medical Association (2013). World Medical Association Declaration of Helsinki: Ethical principles for medical research involving human subjects. JAMA.

[38] Kolmogorov A. (1933). Sulla determinazione empirica di una legge di distribuzione. Inst. Ital. Attuari, Giorn..

[39] Smirnov N.V. (1939). Estimate of deviation between empirical distributions functions in two independent samples. Bull. Mosc. Univ..

[40] Bradley E.L., Kotz E.S., Johnson N.L., Read C.B. (1985). Overlapping coefficient. Encyclopedia of Statistical Sciences.

[41] Golsdtein R. (1994). The overlapping coefficient and an “improved” rank-sum statistic. Stata Techical Bull..

[42] Satorra A., Bentler P.M., von Eye A., Clogg C.C. (1994). Corrections to test statistics and standard errors in covariance structure analysis. Latent Variables Analysis: Applications for Developmental Research.

[43] Boomsma A. (2000). Reporting analyses of covariance structures. Struct. Equ. Model..

[44] Lei P.-W., Wu Q., Hoyle R.H. (2012). Estimation in structural equation modeling. Handbook of Structural Equation Modeling.

[45] Tong X., Bentler P.M. (2013). Evaluation of a new mean scaled and moment adjusted test statistic for SEM. Struct. Equ. Modeling.

[46] Lorenzo-Seva U., ten Berge J.M.F. (2006). Tucker’s congruence coefficient as a meaningful index of factor similarity. Methodology.

[47] Elosua P., Wells C. (2013). Detecting DIF in polytomous items using MACS, IRT and ordinal logistic regression. Psicológica.

[48] Zumbo B.D. (1999). A Handbook on the Theory and Methods of Differential Item Functioning (DIF): Logistic Regression Modeling as a Unitary Framework for Binary and Likert-Type (Ordinal) Item Scores.

[49] Crane P.K., Gibbons L.E., Jolley L., van Belle G. (2006). Differential item functioning analysis with ordinal logistic regression techniques. DIFdetect and difwithpar. Med. Care.

[50] Crane P.K., Gibbons L.E., Narasimhalu K., Lai J.S., Cella D. (2007). Rapid detection of differential item functioning in assessments of health-related quality of life: The functional assessment of cancer therapy. Qual. Life Res..

[51] Crane P.K., van Belle G., Larson E.B. (2004). Test bias in a cognitive test: Differential item functioning in the CASI. Stat. Med..

[52] Cronbach L.J. (1951). Coefficient alpha and the internal structure of tests. Psychometrika.

[53] McDonald R.P. (1999). Test Theory: A Unified Treatment.

[54] Merino-Soto C., Dominguez-Lara S. (2017). Respuesta a carta al editor. Diferenciando la espada de la mano. Rev. Latinoam. Cienc. Soc. Niñez Juv..

[55] Weber R., Popova L. (2012). Testing equivalence in communication research: Theory and Application. Commun. Methods Meas..

[56] Coe R., Soto C.M. (2003). Magnitud del efecto: Una guía para investigadores y usuarios. Rev. Psicol..

[57] Levene H., Olkin I., Ghurye S.G., Hoeffding W., Madow W.G., Mann H.B. (1960). Robust tests for equality of variances. Contributions to Probability and Statistics: Essays in Honor of Harold Hotelling.

[58] Merino-Soto C. (2016). Diferencias entre coeficientes alfa de Cronbach, con muestras y partes pequeñas: Un programa VB. An. Psicol..

[59] Vargas-Halabi T., Mora-Esquivel R. (2017). Sample sizes using structural equation modeling with latent variables: A practical method. Rev. Actual. Investig. Educ..

[60] MacCallum R.C., Browne M.W., Sugawara H.M. (1996). Power analysis and determination of sample size for covariance structure modeling. Psychol. Methods.

[61] MacCallum R., Lee T., Browne M.W. (2010). The issue of isopower in power analysis for tests of structural equation models. Struct. Equ. Model..

[62] Mundfrom D.J., Shaw D.G., Ke T.L. (2005). Minimum sample size recommendations for conducting factor analyses. Int. J. Test..

[63] Wolf E.J., Harrington K.M., Clark S.L., Miller M.W. (2013). Sample size requirements for structural equation models: An evaluation of power, bias, and solution propriety. Educ. Psychol. Meas..

